# Electroacupuncture attenuates high-salt-induced hypertension by modulating neuronal excitability and proteomic signatures in the paraventricular nucleus of mice

**DOI:** 10.3389/fmed.2026.1955943

**Published:** 2026-10-20

**Authors:** Guofan Qiu, Mengyue Fan, Kaixin Li, Bokai Wang, Meng Zhang

**Affiliations:** 1 Institute of Acupuncture and Moxibustion, Shandong University of Traditional Chinese Medicine, Jinan, China; 2 Artemisinin Research Center, and Institute of Chinese Materia Medica, China Academy of Chinese Medical Sciences, Beijing, China; 3 Guangdong Provincial Key Laboratory of Psychiatric Disorders, Guangzhou, China

**Keywords:** ANT1, electroacupuncture, high-salt-induced, hypertension, paraventricular nucleus

## Abstract

**Background:**

High-salt-induced hypertension is associated with sympathetic overactivation, autonomic imbalance, and neurobehavioral abnormalities. The paraventricular nucleus (PVN) regulates autonomic output through projections to the rostral ventrolateral medulla (RVLM) and nucleus tractus solitarius (NTS). Although electroacupuncture (EA) has antihypertensive effects, its central mechanisms remain unclear. Mitochondrial dysfunction contributes to neurogenic hypertension, but the role of mitochondrial regulators such as adenine nucleotide translocator 1 (ANT1/SLC25A4) in PVN dysfunction remains unknown.

**Methods:**

Male C57BL/6J mice were exposed to 2% NaCl for 4 weeks to induce hypertension, followed by EA stimulation at the Taichong (LR3) acupoint for 3 weeks. Blood pressure, behavioral performance, PVN neuronal activity, and molecular alterations were assessed using tail-cuff measurement, behavioral tests, retrograde tracing combined with whole-cell patch-clamp recording, and TMT-based quantitative proteomics, as well as using Western blotting and immunofluorescence analyses.

**Results:**

High-salt exposure increased blood pressure and induced behavioral alterations. EA significantly reduced hypertension and improved behavioral abnormalities. High-salt exposure enhanced the intrinsic excitability of PVN–RVLM and PVN–NTS projection neurons, whereas EA normalized neuronal hyperactivity. Proteomic analysis revealed PVN remodeling involving mitochondrial oxidative phosphorylation, ATP/ADP transport, and transmembrane transport pathways. ANT1/SLC25A4 was downregulated in hypertensive mice but restored by EA, accompanied by reversal of inflammatory and stress-related protein alterations.

**Conclusions:**

EA at LR3 reduced blood pressure and PVN neuronal hyperexcitability in mice with high-salt-induced hypertension. EA was also associated with normalization of mitochondrial-related proteomic changes and ANT1/SLC25A4 expression in the PVN. These findings identify ANT1-associated mitochondrial alterations as a potential molecular correlate of PVN dysfunction during EA treatment, but their causal contribution remains to be determined.

## 1 Introduction

Hypertension is one of the leading risk factors for cardiovascular disease and premature mortality worldwide (1). According to the World Health Organization, approximately 1.3 billion adults aged 30–79 years are living with hypertension globally (2). Despite the availability of multiple antihypertensive therapies, a substantial proportion of patients still fail to achieve adequate blood pressure control (3). Moreover, the latest Global Burden of Disease (GBD) study indicates that excessive dietary sodium intake remains a major contributor to cardiovascular mortality attributable to unhealthy dietary patterns in China (4). Clinical studies further suggest that up to 50% of patients with hypertension exhibit salt sensitivity (5). High-salt-induced hypertension is characterized by excessive sympathetic activation and an increased incidence of cardiovascular and neuropsychiatric complications. Furthermore, accumulating evidence has demonstrated that hypertension is frequently accompanied by anxiety-like behaviors, depression, and cognitive impairment, suggesting that central nervous system dysfunction plays a pivotal role in the development and progression of hypertension.

The paraventricular nucleus (PVN) of the hypothalamus is a critical integrative center regulating autonomic and neuroendocrine functions and plays a central role in maintaining cardiovascular homeostasis and blood pressure regulation. The PVN receives both ascending and descending inputs from multiple brain regions, including the cerebral cortex, limbic system, and brainstem. By integrating signals from peripheral baroreceptors and circulating humoral factors, the PVN coordinates sympathovagal balance and neuroendocrine responses, thereby exerting fine control over the cardiovascular system (6). Anatomically, PVN neurons project directly to the rostral ventrolateral medulla (RVLM) (7) and the nucleus tractus solitarius (NTS) (8), forming key neural pathways involved in the regulation of sympathetic outflow and baroreflex function. Among these pathways, the PVN–RVLM circuit primarily mediates the amplification of excitatory sympathetic drive (9), whereas the NTS plays an essential role in modulating baroreflex sensitivity and suppressing excessive sympathetic activation (10, 11). The balance between these pathways is indispensable for the maintenance of blood pressure homeostasis. Accumulating evidence has demonstrated that the PVN undergoes profound functional remodeling under hypertensive conditions, characterized by increased neuronal excitability, enhanced synaptic transmission, and neurotransmitter imbalance. In particular, hyperexcitability of PVN–RVLM presympathetic neurons markedly augments sympathetic outflow, leading to peripheral vasoconstriction and increased cardiac output, thereby contributing to sustained elevations in blood pressure. Conversely, activation of neurons within the NTS can enhance parasympathetic activity, thereby preserving cardiovascular homeostasis and coordinating behavioral responses (12). Therefore, as a pivotal hub within the central autonomic network, dysfunction of the PVN is considered one of the major central mechanisms underlying neurogenic hypertension. Accordingly, restoring the functional homeostasis of PVN neural circuits and normalizing the activity of the PVN–RVLM and NTS pathways may represent a potential therapeutic strategy for the treatment of hypertension and its associated autonomic dysfunction.

Electroacupuncture (EA), a modern modification of traditional acupuncture, has been increasingly recognized as an effective adjunctive therapy for hypertension. Both experimental and clinical studies have demonstrated that EA can lower blood pressure, suppress sympathetic nerve activity, and ameliorate hypertension-associated emotional and cognitive impairments (13–15). Emerging evidence further suggests that the antihypertensive effects of EA are mediated, at least in part, through central autonomic nuclei, including those located in the hypothalamus and brainstem (16, 17). However, the precise mechanisms by which EA modulates PVN neuronal excitability and alters central autonomic regulation remain incompletely understood.

Mitochondrial dysfunction and oxidative stress have recently been recognized as key pathological mechanisms underlying the development and progression of neurogenic hypertension (18). Within the central autonomic regulatory network, neurons are highly dependent on energy metabolism. Mitochondria not only provide ATP for synaptic transmission and maintenance of membrane potential but also play critical roles in calcium homeostasis and the generation and scavenging of reactive oxygen species (ROS) (2, 19). When mitochondrial function is impaired, insufficient energy supply and enhanced oxidative stress can mutually exacerbate each other, resulting in abnormal neuronal excitability, disrupted synaptic transmission, and autonomic dysregulation. These alterations ultimately promote excessive sympathetic activation and contribute to the sustained elevation of blood pressure (18). Adenine nucleotide translocator 1 (ANT1), also known as solute carrier family 25 member 4 (SLC25A4), is an essential carrier protein located in the inner mitochondrial membrane. ANT1 primarily mediates the exchange of ADP and ATP between the mitochondrial matrix and the cytosol and is therefore a key regulator of mitochondrial oxidative phosphorylation and cellular energy homeostasis. Emerging evidence indicates that ANT1 is involved not only in basal energy metabolism but also in the maintenance of mitochondrial membrane potential, regulation of ROS production, and cellular antioxidant capacity. Aberrant expression or dysfunction of ANT1 can impair the efficiency of the mitochondrial respiratory chain, promote oxidative stress accumulation, and subsequently induce neuronal dysfunction and cellular injury. Consequently, ANT1 has been implicated in the pathogenesis of a variety of neurological and cardiovascular disorders (20, 21).

Despite these advances, the role of ANT1 in neurogenic hypertension remains largely unexplored, particularly within key central cardiovascular regulatory regions such as the PVN. More importantly, it is still unclear whether ANT1 contributes to the initiation and maintenance of PVN neuronal dysfunction during high-salt-induced hypertension and whether it mediates the central antihypertensive effects of electroacupuncture. Therefore, systematically elucidating the role of ANT1 in the PVN autonomic regulatory network will not only advance our understanding of the central mechanisms linking mitochondrial dysfunction to hypertension but may also identify a novel molecular target underlying the therapeutic effects of electroacupuncture in neurogenic hypertension.

## 2 Materials and methods

### 2.1 Experimental animals

A total of 48 male C57BL/6J mice aged 7–8 weeks were used in this study. All animals were purchased from Beijing Vital River Laboratory Animal Technology Co., Ltd. [Animal Production License No. SCXK (Jing) 2021-0006]. The mice were housed in the specific pathogen-free (SPF) animal facility of the Experimental Animal Center of Shandong University of Traditional Chinese Medicine [Animal Use License No. SYXK (Lu) 2022-0009]. Throughout the study, mice had *ad libitum* access to standard chow and water and were maintained under a 12-h light/dark cycle (lights on from 07:00 to 19:00), at an ambient temperature of 20 °C–25 °C and a relative humidity of 40%–70%. Five to six mice were housed per cage. After a 7-day acclimatization period, the experimental procedures were initiated. The experimental protocol was approved by the Experimental Animal Ethics Committee of Shandong University of Traditional Chinese Medicine (approval number: SDUTCM20251015001).

### 2.2 Experimental design

After a 1-week acclimation period, C57BL/6 mice were provided with 2% NaCl solution as the sole source of drinking water for 4 weeks to induce high-salt-induced hypertension. Blood pressure was measured using a CODA non-invasive blood pressure monitoring system. Except for the normal control group, mice with systolic blood pressure ≥140 mmHg and/or diastolic blood pressure ≥80 mmHg, defined according to the 2024 Chinese Guidelines for the Prevention and Treatment of Hypertension, were considered to have developed high-salt-induced hypertension. These hypertensive mice were then randomly assigned to either the hypertension model group or the EA treatment group. Based on Experimental Acupuncture Science, the “Taichong” (LR3) acupoint was selected. Mice were placed in a supine position under isoflurane inhalation anesthesia, with all four limbs gently fixed on a platform. The skin around the acupoint was disinfected with 75% ethanol. Sterile acupuncture needles were inserted perpendicularly into the acupoint and stimulated using a Master-8 pulse stimulator with continuous-wave output at 2 Hz and an intensity of 1 mA for 15 min per session, once daily for 3 weeks. In addition, mice in all groups underwent the same repeated isoflurane anesthesia procedures, whereas only the EA group received electroacupuncture stimulation.

### 2.3 Blood pressure measurement

Blood pressure was measured using the CODA 8 tail-cuff system (Kent Scientific) based on the volume pressure recording (VPR) method. Mice were first acclimated in a quiet environment at 22 °C ± 2 °C for 1 h, followed by pre-warming on a heating pad at 33 °C–35 °C for 5 min to facilitate tail vasodilation. During measurement, the tail cuff was inflated to 250 mmHg and deflated within 20 s. The VPR sensor recorded tail volume changes with a minimum detection threshold of 15 µL. Each measurement session consisted of 10 inflation–deflation cycles. Prior to formal measurements, mice were subjected to tail-cuff training for at least 5 consecutive days to minimize stress-induced variability.

### 2.4 Behavioral tests

#### 2.4.1 Open field test (OFT)

The open field test (XR-XZ301, Shanghai Xinruan Information Technology Co., Ltd.) was used to evaluate spontaneous exploratory behavior, basal locomotor activity, and anxiety-like states in mice. Each mouse was placed individually into a square open field arena (40 cm × 40 cm × 40 cm) and allowed to freely explore for 15 min. Locomotor activity was recorded using an automated tracking system, including total distance traveled, average speed, time spent in the central zone, and number of entries into the central zone. In general, healthy C57BL/6 mice exhibit moderate exploratory behavior, with a total travel distance of approximately 15–30 m and central zone occupancy of about 20%–40%. In contrast, animals with psychiatric disorder-like or anxiety-like phenotypes typically show two characteristic patterns: hyperactivity (increased total distance and excessive central exploration), reflecting impaired inhibitory control or mania-like behavior; or hypoactivity and anxiety-like behavior (reduced total distance, central zone occupancy <10%, and increased thigmotaxis), indicating avoidance behavior or reduced motivational drive. All tests were conducted under low-light and low-noise conditions and were separated from other behavioral assays by sufficient intervals to avoid interference. After each test, the apparatus was cleaned with ethanol to eliminate olfactory cues.

#### 2.4.2 Elevated plus maze (EPM)

The elevated plus maze (XR-XZ302, Shanghai Xinruan Information Technology Co., Ltd.) was used to assess anxiety-like behavior in mice. The apparatus consisted of two open arms (30 cm × 5 cm) and two closed arms (30 cm × 5 cm × 15 cm), with a central platform (5 cm × 5 cm), elevated 50 cm above the floor. The maze was constructed of opaque resin to minimize reflection and external interference. At the start of the test, each mouse was placed in the central platform facing an open arm and allowed to freely explore for 5 min. Behavior was recorded using a video tracking system. Parameters included the number of entries into open and closed arms, time spent in each arm, and total distance traveled. An entry was defined as all four paws entering a given arm. Under normal conditions, C57BL/6J mice display moderate exploration of the open arms, with open-arm time accounting for approximately 20%–40% of total test duration, reflecting a balance between exploration and risk avoidance. Anxiety-like models typically show reduced open-arm exploration, whereas certain neuropsychiatric models may exhibit increased open-arm activity, indicating impaired risk assessment or impulsivity. All experiments were conducted under low-light conditions (20–30 lux) with minimal noise (<50 dB). The apparatus was thoroughly cleaned with 75% ethanol and air-dried between trials to eliminate olfactory cues.

#### 2.4.3 Forced swimming test (FST)

The forced swimming test was used to evaluate depression-like behavior and stress-coping strategies in mice. Each mouse was placed in a transparent cylindrical container (height 25 cm, diameter 20 cm) filled with water to a depth of approximately 15 cm, maintained at 23 °C–25 °C to prevent tail contact with the bottom and minimize temperature-induced stress. Each mouse was allowed to swim freely for 6 min, and behavior was recorded throughout. The first 2 min were considered habituation and excluded from analysis, while the last 4 min were used for behavioral scoring. The primary parameter was immobility time, defined as the absence of active escape-oriented behaviors while the animal remained afloat with minimal movement. In general, C57BL/6J mice display relatively short immobility durations, maintaining active swimming and climbing behaviors. In contrast, depression-like or neuropsychiatric model mice exhibit significantly prolonged immobility, reflecting behavioral despair and reduced active coping capacity. After testing, mice were immediately dried with towels and placed in a warm environment for recovery to prevent hypothermia. All experiments were performed at the same time of day to minimize circadian influences.

#### 2.4.4 Novel object recognition test

The novel object recognition test was used to assess recognition memory and exploratory preference for novelty, which depend on the integrity of hippocampal function. The experiment was conducted in a square open field arena (40 cm × 40 cm) and consisted of three phases: habituation, familiarization (training), and testing. During habituation, mice were individually placed in the empty arena and allowed to explore freely for 5–10 min to reduce novelty-induced anxiety. After 24 h, two identical objects (e.g., blocks or glass bottles) were placed symmetrically in the arena during the familiarization phase, and mice were allowed to explore for 5–10 min. After a defined retention interval (e.g., 1 h for short-term memory or 24 h for long-term memory), one familiar object was replaced with a novel object differing in shape, material, or color. Mice were then reintroduced into the arena for the same duration, and behavior was recorded by an overhead camera. Exploration was defined as the mouse directing its nose toward the object at a distance of <2 cm, including sniffing or touching, but excluding sitting on or climbing the object. Key variables included time spent exploring novel versus familiar objects. To minimize confounding factors, all objects were stable and non-climbable, and were thoroughly cleaned with 75% ethanol between trials. Object locations were counterbalanced across animals. All tests were conducted under consistent lighting and noise conditions during the same time of day to minimize circadian effects.

### 2.5 Retrograde labeling of PVN-RVLM and PVN-NTS neurons

Mice were anesthetized with inhaled isoflurane (3% for induction and 1%–2% for maintenance) and placed in a stereotaxic apparatus (RWD Life Science, Shenzhen, China). Anesthesia was maintained throughout the surgical procedure. Body temperature was maintained throughout the surgical procedure using a thermostatically controlled heating pad. After fixation of the head, the skull was exposed and adjusted to ensure that the bregma and lambda were positioned in the same horizontal plane. According to the Mouse Brain Atlas, stereotaxic coordinates were used to target the NTS and the RVLM. For RVLM injections, the coordinates were 6.6–6.8 mm posterior to bregma, 1.0 mm lateral to the midline, and 4.5–4.8 mm ventral from the dural surface. For NTS injections, the coordinates were 7.48 mm posterior to bregma, 0.40 mm lateral to the midline, and 4.20 mm ventral from the dural surface. A Hamilton microsyringe connected to a microinfusion pump was loaded with 100 µL R180 Red Retrobeads™ IX (Lumafluor Inc., Naples, FL, USA) and slowly advanced into the designated brain region. The tracer was delivered at a controlled infusion rate to minimize tissue damage and backflow. After injection, the needle was maintained in position for 10 min to allow tracer diffusion and was then slowly withdrawn. The incision was closed, and mice were allowed to recover on a warming pad until fully awake. Retrograde labeling was allowed to develop for 7 days before subsequent electrophysiological experiments.

### 2.6 Technique of whole-cell patch-clamp recording in acute brain slices

(1) Preparation of acute PVN brain slices: Mice were deeply anesthetized with an intraperitoneal injection of pentobarbital (50 mg/kg). After confirming deep anesthesia by the absence of reflex responses, mice were transcardially perfused with an ice-cold sucrose-containing slicing solution (in mM: sucrose 220, KCl 2.5, NaH2PO4 1.25, MgSO_4_ 2, NaHCO_3_ 26, glucose 25, CaCl_2_ 1, ascorbic acid 0.4, pyruvate 2.0) bubbled with carbogen. Following perfusion, animals were euthanized by rapid decapitation, and the brains were immediately removed. Coronal slices (250 μm) containing the PVN were cut using a vibratome (Leica VT1200s, Leica Microsystems, Buffalo Grove, IL, USA). Sectioning was performed in ice-cold oxygenated artificial cerebrospinal fluid (ACSF) of the following composition (in mM): NaCl 119, KCl 2.5, MgSO_4_ 1.26, NaHCO_3_ 26, NaH₂PO_4_ 1.25, glucose 20, ascorbic acid 0.4, CaCl_2_ 2.0, pyruvate 2.0; pH 7.3; osmolarity 295–305 mOsmol/L. After cutting, slices were transferred to a holding chamber containing ACSF and maintained at room temperature (22 °C) until use. During recording, the slice was placed in a recording chamber and continuously superfused with carbogen-bubbled ACSF at a flow rate of 3 mL/min, with the temperature maintained at 32 °C. (2) Whole-cell recording of spontaneous excitability: Fluorescently labeled neurons within the PVN region were selected for whole-cell recordings. A potassium gluconate-based internal solution was used (in mM: K-gluconate 135, EGTA 0.2, HEPES 10, KCl 10, MgCl_2_ 0.9, Mg-ATP 4, Na-GTP 0.3, phosphocreatine 20; pH 7.2–7.3; osmolarity 270–275 mOsmol/L). Cell sealing and membrane rupture were performed in voltage-clamp mode. For electrophysiological recordings, neurons were excluded from analysis if the recording was unstable or failed to meet the required recording-quality criteria. Exclusion criteria included an unstable resting membrane potential, substantial changes in series resistance during recording, marked instability of input resistance, excessive recording noise, loss of the whole-cell configuration, or failure to complete the recording protocol. Action potentials (APs) were then recorded in current-clamp mode by applying incremental step currents from +0 pA to +150 pA in 10-pA increments, with each step 500 ms. Action potential firing was observed and recorded throughout the entire protocol.

### 2.7 PVN tissue collection and TMT-based proteomic analysis.

Following deep anesthesia with pentobarbital (50 mg/kg, i.p.), mice were euthanized by rapid decapitation. The brains were rapidly removed, and tissues were immediately frozen for subsequent proteomic and molecular analyses. Coronal brain sections were continuously prepared using a cryostat microtome with a thickness of 100 μm. According to the stereotaxic atlas of the mouse brain, the PVN of the hypothalamus was rapidly identified and located within the coordinate range approximately 0.6–1.2 mm posterior to the bregma. Under frozen conditions, bilateral PVN tissues were precisely microdissected from the brain sections using pre-cooled biopsy punches. The entire dissection procedure was performed under strictly controlled low-temperature conditions (approximately −20 °C) to minimize protein degradation. The collected PVN samples were immediately transferred into pre-chilled microcentrifuge tubes and stored at −80 °C until further analysis. For protein extraction, PVN tissues were homogenized in ice-cold RIPA lysis buffer supplemented with protease and phosphatase inhibitor cocktails. After incubation on ice, lysates were centrifuged, and the supernatants were collected for subsequent protein quantification. Protein concentrations were determined using the bicinchoninic acid (BCA) assay. Extracted proteins were subjected to reduction, alkylation, and enzymatic digestion to generate peptide fragments. The resulting peptides were labeled with tandem mass tags (TMT) and analyzed by high-resolution mass spectrometry. TMT-based quantitative proteomics was employed for high-throughput protein identification and relative quantification among experimental groups. Finally, bioinformatics analysis was performed using R software (version 4.4.2; R Foundation for Statistical Computing, Vienna, Austria) to process the proteomic datasets and identify biologically relevant differentially expressed proteins and functional alterations.

### 2.8 Western blotting

The PVN tissues were rapidly dissected and homogenized in RIPA lysis buffer supplemented with protease and phosphatase inhibitor cocktails. The lysates were incubated on ice and centrifuged at 12,000 × g for 15 min at 4 °C. The supernatants were collected, and protein concentrations were determined using a bicinchoninic acid (BCA) protein assay kit. Equal amounts of total protein from each sample were mixed with loading buffer and denatured at 95 °C for 5 min.

The protein samples were separated by SDS-PAGE and subsequently transferred onto polyvinylidene difluoride (PVDF) membranes. After blocking with 5% non-fat milk in Tris-buffered saline containing 0.1% Tween-20 (TBST) for 1 h at room temperature, the membranes were incubated overnight at 4 °C with primary antibodies against ANT1/SLC25A4 (rabbit, Abclonal, A15027, 1:2,000). After washing three times with TBST, the membranes were incubated with HRP-conjugated secondary antibodies for 1 h at room temperature. Protein bands were visualized using an enhanced chemiluminescence (ECL) detection system. The band intensities were quantified using ImageJ software. ANT1/SLC25A4 protein expression was normalized to the corresponding loading control, and the relative protein expression levels were calculated for each experimental group.

### 2.9 Immunofluorescence (IF) staining

Following deep anesthesia with pentobarbital (50 mg/kg, i.p.), mice were transcardially perfused with phosphate-buffered saline (PBS), followed by 4% paraformaldehyde (PFA). The brains were removed, post-fixed in 4% PFA, and then cryoprotected in sucrose solution. Coronal brain sections containing the PVN of the hypothalamus were prepared using a cryostat. The sections were incubated overnight at 4°C with primary antibodies against ANT1/SLC25A4 (rabbit, Abclonal, A15027, 1:200), GFAP (mouse, Abcam, ab190288, clone 5C10, 1:500), and IBA1 (Thermo Fisher Scientific, MA550413, 1:200). After washing with PBS, the sections were incubated with appropriate species-specific fluorescent secondary antibodies for 1 h at room temperature in the dark. Nuclei were counterstained with DAPI. The sections were then washed, mounted with an anti-fade mounting medium, and coverslipped. Images of the PVN were acquired using a confocal microscope under identical acquisition settings for all experimental groups. For quantitative analysis, regions of interest (ROIs) encompassing the PVN were defined according to a mouse brain atlas. Fluorescence intensity and the colocalization of ANT1 with GFAP or IBA1 were analyzed using ImageJ software.

### 2.10 Statistical analysis

The statistical analysis was applied by GraphPad Prism 9 software (San Diego, California, USA). Data were represented as the mean ± SE. One-way analysis of variance was performed by comparing differences between groups. If *F*-values showed significance, Tukey’s *post hoc* test was used. *P* < 0.05 means a statistically significant difference. To minimize potential observer bias, investigators performing the behavioral, electrophysiological, and molecular analyses were blinded to the experimental group assignments during data acquisition and analysis.

## 3 Results

### 3.1 Chronic 2% NaCl administration induces hypertension and behavioral alterations in mice

To evaluate the effects of a high-salt diet on cardiovascular function in C57BL/6 mice, systolic blood pressure (SBP), diastolic blood pressure (DBP), and heart rate (HR) were continuously monitored for 4 weeks (Figures 1A–E). At baseline (Week 0), SBP levels in both the control and model groups were within the normal physiological range, with no statistically significant difference between groups. After administration of a 2% NaCl diet, SBP in the model group exhibited a time-dependent progressive increase. At Week 2, SBP began to show an upward trend. By Weeks 3 and 4, SBP further increased to approximately 136.55 ± 2.76 mmHg and 142.32 ± 2.88 mmHg, respectively, which were significantly higher than those of the control group (*P* < 0.0001), showing extremely significant differences. A similar trend was observed for DBP. In the model group, DBP began to diverge from the control group at Week 2. By the end of the experiment (Week 4), DBP in the model group remained at a markedly elevated level, whereas the control group maintained a stable level (*P* < 0.0001). Notably, despite the significant fluctuations in blood pressure, heart rate remained within the normal physiological range (440–500 bpm) throughout the 4-week experimental period in both groups (Figure 1D) (*P* > 0.05). In addition, body weight analysis showed that high-salt intake significantly increased blood pressure but did not promote weight gain (Figure 1E) (*P* > 00.05). Overall, the 2% NaCl diet successfully established a hypertension model in C57BL/6J mice.

**Figure 1:**
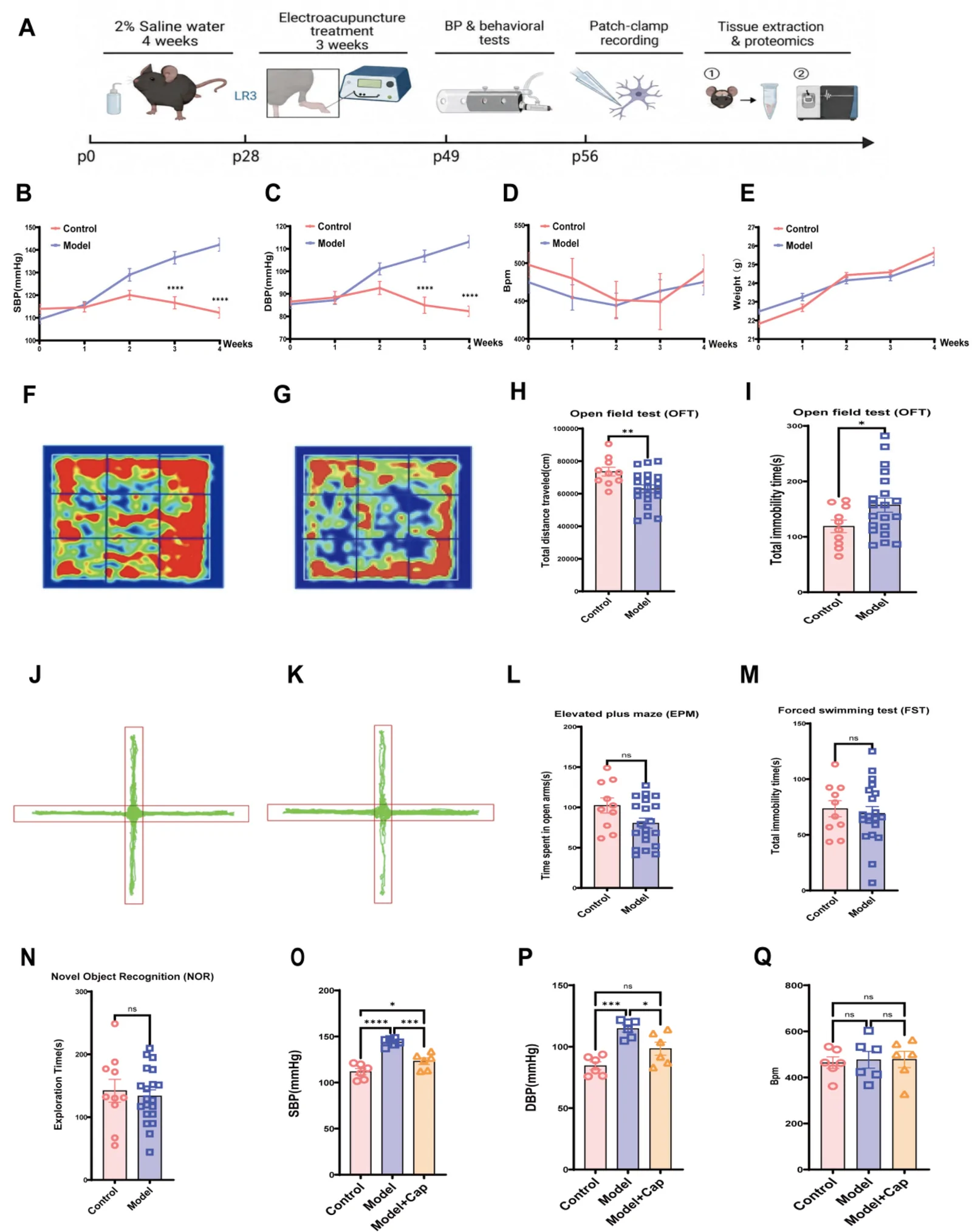
High-salt diet induces hypertension and neurobehavioral changes in mice. **(A)** Experimental timeline and study design. **(B–E)** Longitudinal physiological monitoring over 4 weeks of high-salt interventions (*n* = 10 for Control, *n* = 20 for Model), showing systolic blood pressure (SBP) **(B)**, diastolic blood pressure (DBP) **(C)**, heart rate (bpm) **(D)**, and body weight trajectory **(E)**. **(F–I)** Evaluation of locomotor activity and anxiety-like behavior in the open field test (OFT; *n* = 10 for Control, *n* = 20 for Model), including representative heatmap movement tracks for Control **(F)** and Model **(G)** groups, total distance traveled **(H)**, and total immobility time **(I)**. **(J–L)** Assessment of anxiety-like behaviors in the elevated plus maze (EPM; *n* = 10 for Control, *n* = 20 for Model), showing representative movement trajectories for Control **(J)** and Model **(K)** groups, and total time spent in open arms **(L)**. **(M)** Total immobility time in the forced swimming test (FST; *n* = 10 for Control, *n* = 20 for Model). **(N)** Total exploration time in the novel object recognition (NOR) test (*n* = 10 for Control, *n* = 20 for Model). **(O–Q)** Blood pressure and heart rate responses across treatment groups (*n* = 6 mice per group), showing SBP **(O)**, DBP **(P)**, and heart rate **(Q)**. Data are presented as mean ± SEM [*n* = 10 for Control group, *n* = 20 for Model group in panels **(B–N)**; *n* = 6 mice per group in panels **(O–Q)**]. *p < 0.05, **p < 0.01, ***p < 0.001, ****p < 0.0001; ns, not significant.

Previous studies have reported that hypertension is associated with anxiety, agitation, memory impairment, depression, and dementia-like phenotypes (22–24). To systematically evaluate behavioral alterations in salt-sensitive hypertensive mice, a series of behavioral tests were conducted after successful model establishment. In the open field test (OFT) (Figures 1F–I), the model group exhibited a significant reduction in total travel distance compared with the control group (*P* < 0.01), accompanied by a significant increase in total immobility time (*P* < 0.001). These findings indicate a marked reduction in spontaneous locomotor activity and the presence of behavioral inhibition, consistent with previous studies. Notably, no statistically significant differences were observed between groups in the forced swimming test (FST), elevated plus maze (EPM), or novel object recognition (NOR) test (*P* > 0.05) (Figures 1J–N). Although the open-arm time in the EPM showed a decreasing trend in the model group, it did not reach statistical significance (*P* = 0.0665).

To validate the reliability of the high-salt-induced hypertension model, captopril was used as a positive control. Compared with the control group (Figures 1O–Q), the model group exhibited significantly increased systolic blood pressure (SBP) and diastolic blood pressure (DBP) (*P* < 0.05), whereas captopril treatment markedly reduced both SBP and DBP compared with the model group (*P* < 0.05), confirming the responsiveness of the model to antihypertensive intervention. No significant differences in heart rate (HR) were observed among the three groups (*P* > 0.05). These results demonstrate the successful establishment of the high-salt-induced hypertension model and its pharmacological validity.

These results suggest that 4 weeks of high-salt intervention significantly impaired basal locomotor activity in mice, but did not induce pronounced despair-like behavior, anxiety-like behavior, or short-term recognition memory deficits. This indicates that the model primarily reflects a psychomotor retardation-related dimension of hypertension-associated behavioral alterations rather than broad affective or cognitive dysfunction.

### 3.2 Electroacupuncture at taichong (LR3) alleviates hypertension and neurobehavioral impairments in high-salt-induced hypertension mice

The Taichong (LR3) acupoint, located on the dorsum of the foot in the depression between the first and second metatarsal bones, is one of the most frequently used acupoints for the clinical management of hypertension (25). Based on its established antihypertensive efficacy, bilateral Taichong (LR3) acupoints were selected for EA intervention in salt-sensitive hypertensive mice. EA treatment was administered once daily for 3 consecutive weeks.

After EA treatment (Figures 1A–C), stimulation at Taichong acupoint exhibited a pronounced antihypertensive effect and effectively reversed the high-salt-induced elevation in blood pressure. In the model group, SBP continuously increased over 3 weeks and reached a peak at Week 3. In contrast, in the model + EA group, the upward trend was attenuated as early as Week 1 and gradually decreased thereafter. By Week 3, SBP in the EA-treated group was significantly lower than that in the model group (137.07 ± 4.89 mmHg). Notably, although SBP in the model + EA group remained different from that in the control group, a clear therapeutic effect was observed. A similar trend was observed for diastolic blood pressure. DBP in the model group increased steadily over time, whereas EA treatment effectively suppressed this increase. At Week 3, DBP in the model + EA group was significantly lower than that in the model group. In addition, heart rate in all three groups fluctuated within the range of 400–500 bpm over the 21-day period, with no significant differences among groups. This suggests that neither the 2% saline-induced model nor EA intervention caused pathological disturbances in cardiac rhythm, and the antihypertensive process was relatively stable.

Subsequently, open field test (OFT) was performed across all three groups (Figures 2D,E). The results showed that the model group exhibited a significant reduction in total locomotor distance compared with the control group (*P* < 0.05), accompanied by a significant increase in total immobility time (*P* < 0.01). Importantly, after EA intervention, immobility time in the model + EA group was significantly reduced compared with the model group (*P* < 0.05) and recovered to a level comparable with the control group. Although total locomotor distance in the EA-treated group showed an increasing trend compared with the model group, it did not reach statistical significance. Taken together, these findings indicate that EA intervention effectively alleviated model-induced behavioral inhibition, enhanced exploratory motivation, and promoted normalization of overall behavioral performance.

**Figure 2:**
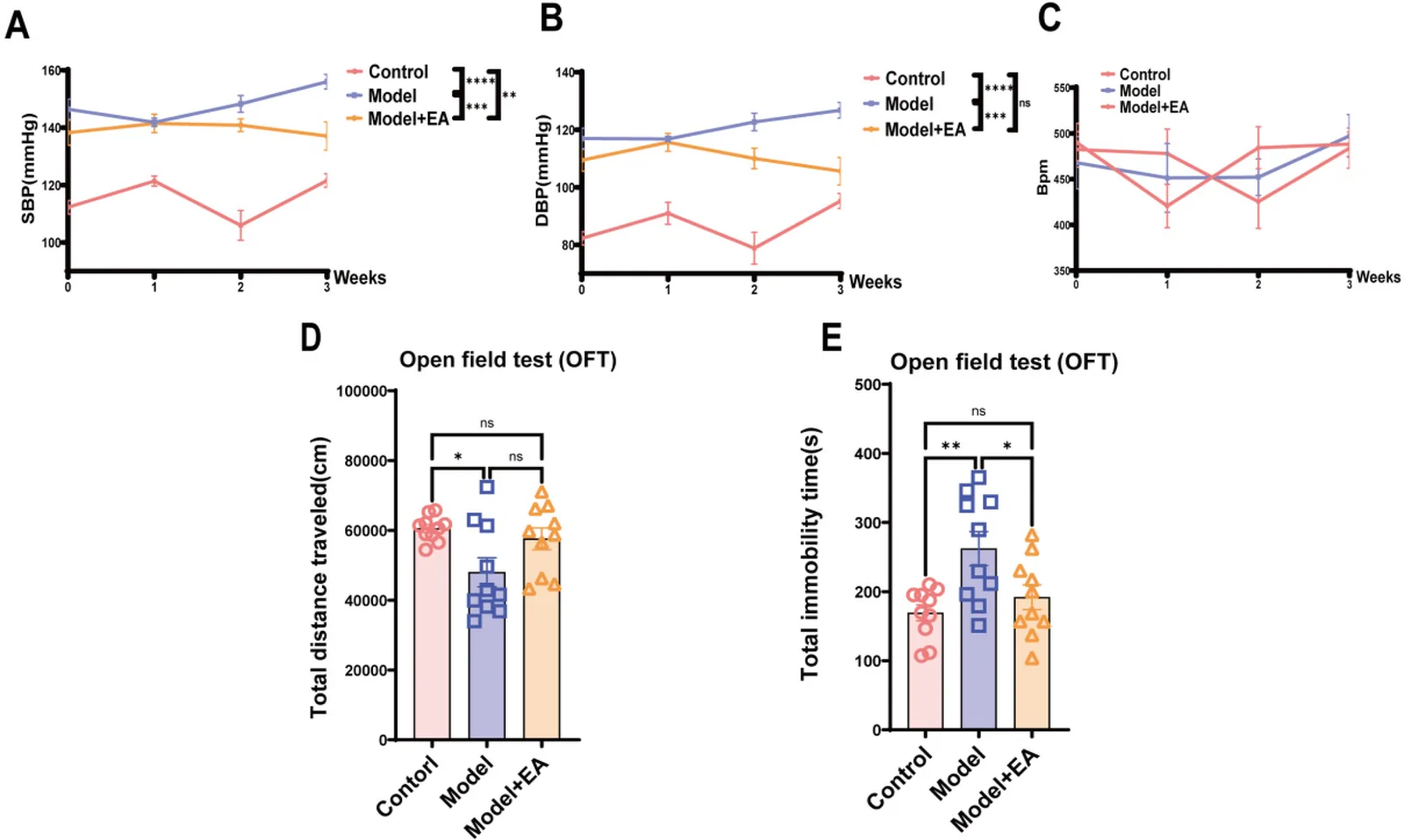
Effects of electroacupuncture (EA) on blood pressure, heart rate, and behavioral changes in mice. **(A–C)** Dynamic changes in systolic blood pressure (SBP, A), diastolic blood pressure (DBP, B), and heart rate (Bpm, C).**(D,E)** Assessment of anxiety- and depression-like behaviors using the Open Field Test (OFT), including total distance traveled (cm) **(D)** and total immobility time (s) **(E)**. Data are presented as mean ± SEM. *p < 0.05, **p < 0.01, ***p < 0.001, ****p < 0.0001; ns, not significant.

### 3.3 Electroacupuncture remodels the excitability of PVN-RVLM neurons in high-salt-induced hypertension mice

In the previous section, we demonstrated that EA stimulation at the Taichong (LR3) acupoint significantly reduced blood pressure in high-salt-induced hypertensive mice and improved behavioral abnormalities, including decreased locomotor activity in the open field test. However, the central neural mechanisms underlying the antihypertensive effects of EA remain unclear. Previous studies have shown that hypertension is closely associated with enhanced neuronal excitability within central autonomic regulatory circuits, particularly in the hypothalamic PVN and RVLM, which play critical roles in sympathetic regulation and blood pressure control (26). Therefore, we hypothesized that EA at the Taichong acupoint may contribute to blood pressure regulation by modulating the abnormal excitability of PVN–RVLM projection neurons. To test this hypothesis, whole-cell patch-clamp recordings were performed to evaluate spontaneous firing activity and intrinsic electrophysiological properties of PVN neurons projecting to the RVLM in high-salt-induced mice following EA intervention.

Retrograde tracing was performed by injecting red fluorescent retrobeads into the RVLM of mice (Figure 3A). Under fluorescence microscopy, retrogradely labeled neurons were successfully identified in the PVN. Subsequent whole-cell patch-clamp recordings were performed specifically on these labeled neurons. Electrophysiological recordings revealed that there were no significant differences among the control, model, and EA groups in intrinsic passive membrane properties, including resting membrane potential, membrane resistance, and membrane capacitance (Figures 3B–D). These results suggest that neither modeling nor EA intervention altered the passive membrane properties of PVN neurons. However, in terms of functional neuronal output, PVN neurons in the model group exhibited a significantly increased firing frequency in response to identical step current injections (0–150 pA) compared with the control group (Figures 3E,F). This increase in action potential firing frequency directly indicates a pathological enhancement of intrinsic neuronal excitability induced by the hypertensive model. In contrast, EA treatment significantly reduced the firing frequency compared with the model group, demonstrating a clear inhibitory effect of EA on PVN neuronal hyperexcitability. Furthermore, analysis of single action potential waveforms showed no significant differences among the three groups in firing threshold, action potential amplitude, afterhyperpolarization (AHP), half-width, rising slope, and decay slope (Figures 3G–L).

**Figure 3:**
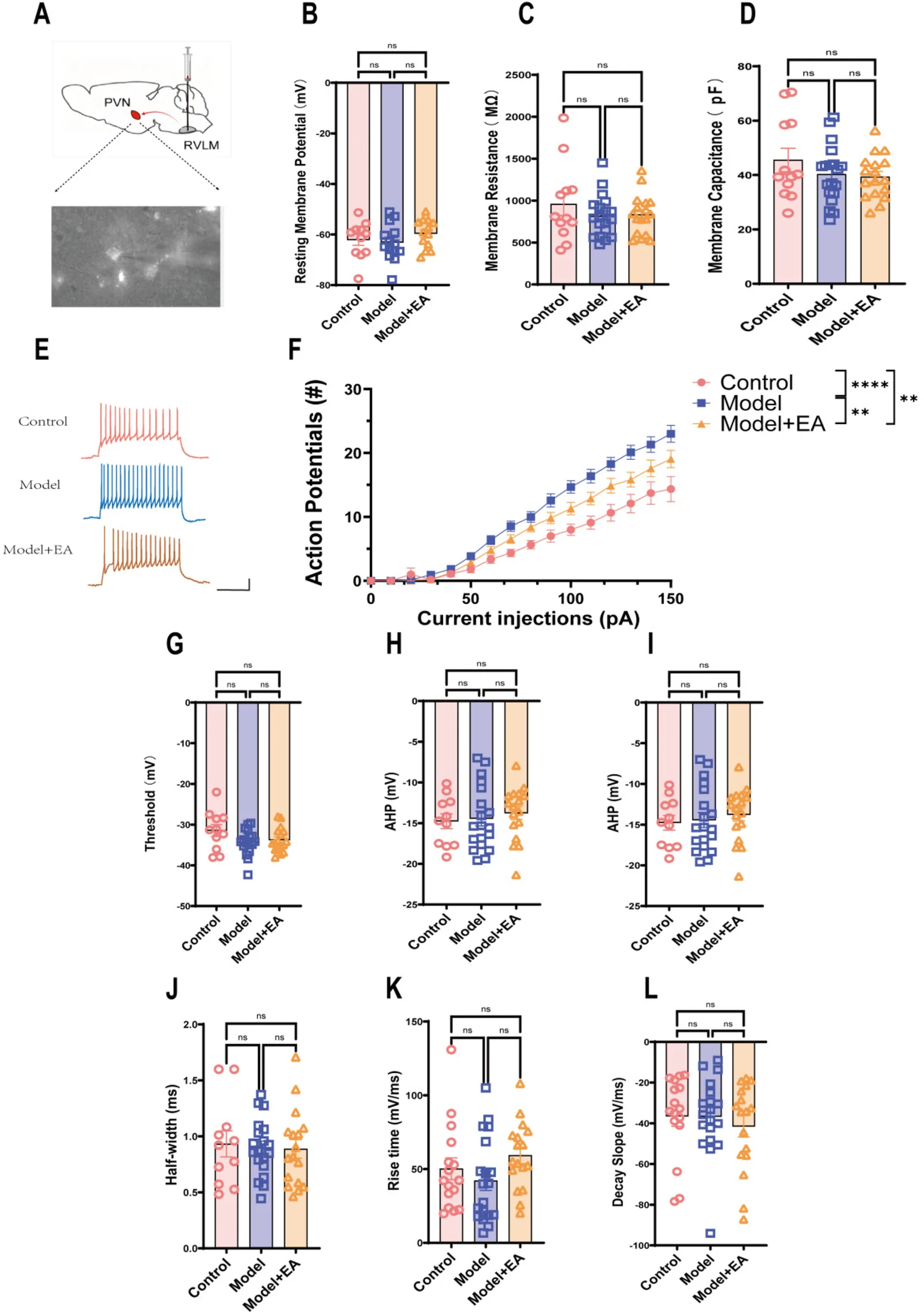
Effects of electroacupuncture at LR3 on the excitability of PVN-RVLM neurons in salt-sensitive hypertensive mice. **(A)** Representative diagrams of neuronal retrograde tracing and whole-cell patch-clamp recording of PVN-RVLM projecting neurons. **(B,C)** Resting membrane potential, membrane resistance, and membrane capacitance of PVN-RVLM neurons across groups. **(E)** Representative action potential traces evoked by a + 150 pA current injection in each group. **(F)** Frequency–current **(F–I)** curves showing the number of action potentials (spikes) in PVN-RVLM neurons. **(G–L)** Quantitative analysis of action potential properties in PVN-RVLM neurons, including threshold **(G)**, amplitude **(H)**, afterhyperpolarization (AHP) amplitude **(I)**, half-width **(J)**, rise slope **(K)**, and decay slope **(L)**. Data are presented as mean ± SEM (*n* = 11 for Control group, *n* = 19 for Model group, *n* = 17 for Model + EA group). *p < 0.05, *p* < 0.01, ***p < 0.001, ****p < 0.0001.

In summary, the hypertensive model induced a pathological hyperexcitable firing phenotype in PVN neurons, whereas EA treatment effectively reversed this abnormality by reducing neuronal firing frequency, thereby restoring intrinsic excitability toward normal levels.

### 3.4 Electroacupuncture remodels the excitability of PVN-NTS neurons in high-salt-induced hypertension mice

We observed that the excitability of PVN–RVLM projection neurons was significantly increased in high-salt-induced hypertensive mice, whereas EA effectively attenuated this abnormal hyperexcitability. Autonomic imbalance is recognized as a critical contributor to the pathogenesis and progression of hypertension (27). The PVN of the hypothalamus serves as a pivotal integrative center for central autonomic regulation (6). Hyperactivity of PVN neurons projecting to the RVLM has been identified as a major driver of excessive sympathetic outflow and the development of neurogenic hypertension (28, 29). In contrast, the NTS, as the primary integrative center of the parasympathetic nervous system, plays an essential role in cardiovascular homeostasis through the regulation of vagal activity and autonomic balance (30). We hypothesized that the neuronal hyperexcitability of the PVN observed under hypertensive conditions may not be restricted to PVN neurons projecting to the RVLM, but may also extend to PVN neurons projecting to the NTS. To test this hypothesis, whole-cell patch-clamp recordings combined with retrograde tracing were performed to selectively identify and record PVN neurons projecting to the NTS (PVN–NTS projection neurons) in a mouse model of high-salt-induced hypertension. We then investigated the effects of EA stimulation at the Taichong acupoint (LR3) on the spontaneous firing activity and intrinsic excitability of PVN–NTS projection neurons, thereby characterizing the electrophysiological changes in this population of PVN projection neurons and their modulation by EA.

To explore the electrophysiological properties of PVN neurons projecting to the nucleus tractus solitarius in high-salt-induced hypertensive mice, whole-cell patch-clamp recordings were performed across the control, model, and model + EA groups. First, in terms of intrinsic passive membrane properties (Figures 4A–D), no significant differences were observed among the three groups in resting membrane potential, membrane resistance, or membrane capacitance, indicating that neither high-salt-induced hypertension nor EA intervention significantly altered the passive membrane properties of PVN–NTS projecting neurons.

**Figure 4:**
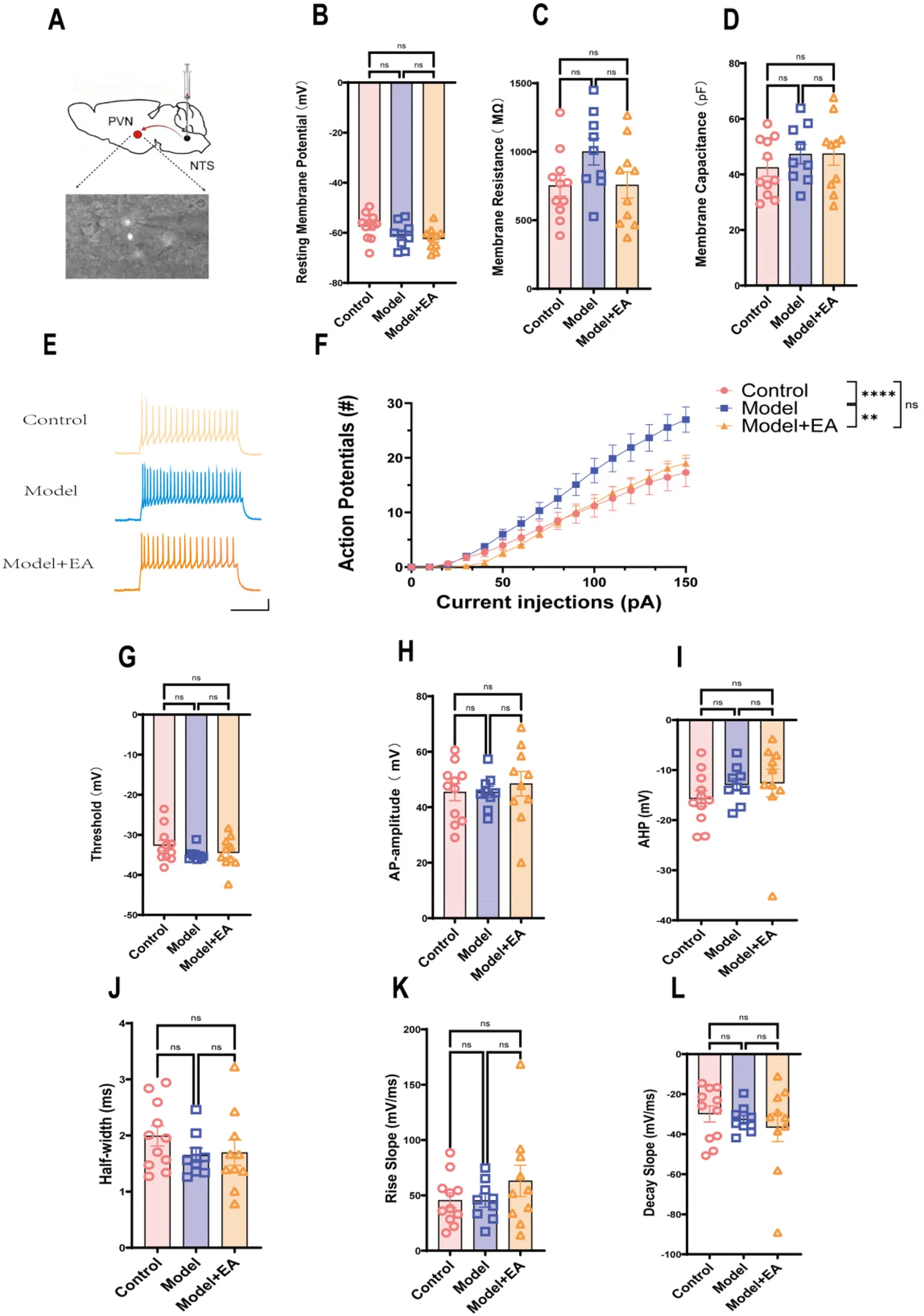
Effects of electroacupuncture at LR3 on the excitability of PVN-NTS neurons in salt-sensitive hypertensive mice. **(A–C)** Resting membrane potential **(A)**, membrane resistance **(B)**, and membrane capacitance **(C)** of PVN-NTS neurons across groups. **(D)** Representative action potential traces evoked by a + 150 pA current injection in each group. **(E)** Frequency–current ($F-I$) curves showing the number of action potentials (spikes) in PVN-NTS neurons. **(F–K)** Quantitative analysis of action potential properties in PVN-NTS neurons, including threshold **(F)**, amplitude **(G)**, afterhyperpolarization (AHP) amplitude **(H)**, half-width **(I)**, rise slope **(J)**, and decay slope **(K)**. Data are presented as mean ± SEM (*n* = 11 for Control group, *n* = 9 for Model group, *n* = 10 for Model + EA group). Data are presented as mean ± SEM. *p < 0.05, **p < 0.01, ***p < 0.001, ****p < 0.0001; ns, not significant.

Regarding neuronal excitability (Figures 4E,F), representative firing curves showed that neurons in the model group generated more action potentials under identical current injections compared with the control group. Quantitative analysis demonstrated that, with increasing current steps, the firing frequency in the model group was significantly higher than that in the control group, indicating enhanced neuronal excitability. In contrast, firing frequency in the model + EA group was markedly reduced compared with the model group and approached control levels, suggesting that EA effectively suppressed hyperexcitability in this pathway. Further analysis of action potential waveform parameters (Figures 4G–L), including firing threshold, action potential amplitude, afterhyperpolarization (AHP), half-width, rising slope, and decay slope, revealed no significant differences among the three groups. These findings suggest that high-salt-induced hypertension does not significantly alter the waveform characteristics of single action potentials, and its primary effect is reflected in the regulation of overall neuronal firing frequency.

In summary, high-salt-induced hypertension significantly enhances the excitability of PVN neurons projecting to the NTS, whereas electroacupuncture intervention effectively reverses this abnormal hyperexcitable state.

### 3.5 Effects of electroacupuncture at taichong (LR3) on the PVN proteomic profile in high-salt-induced hypertension

The neuronal excitability of PVN neurons projecting to both the RVLM and the NTS was significantly enhanced, whereas EA intervention effectively suppressed this abnormal hyperexcitability. These findings suggest that the PVN plays a pivotal role in maintaining the balance between sympathetic and parasympathetic activity. An important question is whether EA exerts its inhibitory effects primarily through modulation of this key brain region. To address this, proteomic profiling of the PVN was performed to systematically investigate molecular changes underlying the therapeutic effects of EA in hypertension.

Using high-throughput proteomics, we quantitatively analyzed the PVN proteome in the control, salt-induced hypertensive, and EA-treated groups. Volcano plot analysis (Figures 5A,B) revealed multiple differentially expressed proteins between the model and control groups, with significantly more downregulated than upregulated proteins. Mitochondrial function and oxidative phosphorylation-related proteins, including Cox7b, Vdac1, Uqcrb, Uqcrc2, and Atp5pb, were markedly downregulated. In addition, neuronal structural and functional proteins such as Thy1, Slc6a11, Tubb5, and Basp1 were also reduced. Conversely, inflammation- and stress-related proteins, including S100b, Mif, Ppp3r1, Cfl1, Ncdn, Gda, and Ptgds, were significantly upregulated, with Ptgds showing the strongest increase, whereas Cox7b and Vdac1 exhibited the most pronounced decrease. In the EA versus control comparison, upregulated proteins included Araf, Gfap, Arl9, Sh3bgrl3, Arpc3, Ppp3cb, Psmb1, Pafah1b2, Hnrnpa2b1, and Mif, whereas downregulated proteins included Basp1, Tmsb4x, S100b, Gap43, Lman1, Slc6a11, Thy1, Cfl1, Pea15, and Ptk2. Notably, Araf and Gfap showed the most prominent upregulation, while Basp1, Tmsb4x, and S100b were most significantly reduced. Further comparative analysis (Figure 5C) showed that 10 proteins downregulated in the model group were upregulated by EA, while six proteins upregulated in the model group were downregulated after EA intervention. This indicates that EA selectively reverses disease-associated proteomic alterations.

**Figure 5:**
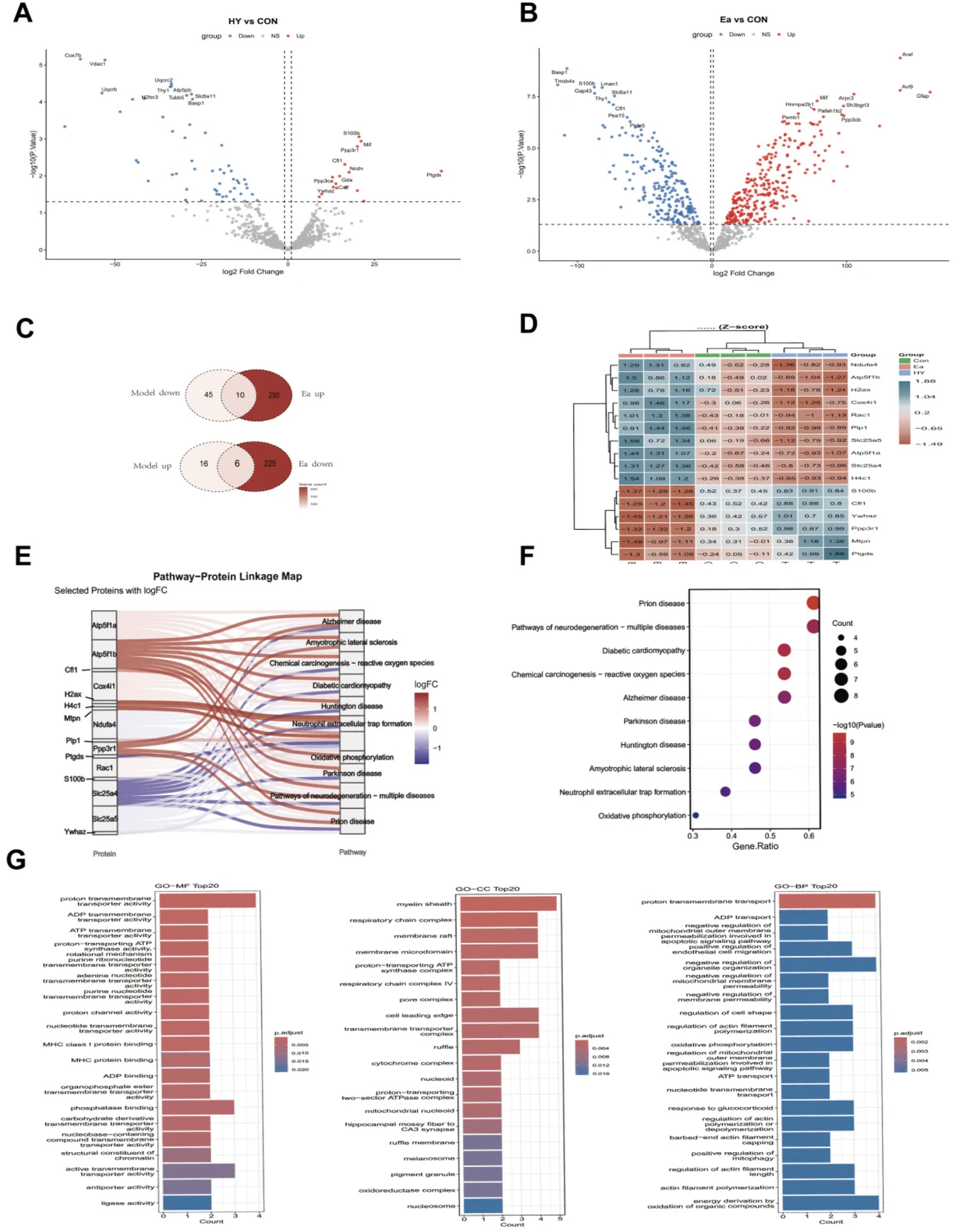
Proteomic analysis of differentially expressed proteins and functional enrichment pathways in the brain. **(A,B)** Volcano plots showing differentially expressed proteins (DEPs) between the HY (model) and CON groups **(A)**, as well as between the EA (treatment) and CON groups **(B)**, with red dots representing upregulated proteins and blue dots representing downregulated proteins (|log_2 FC|> 0.58, *p* < 0.05). **(C)** Venn diagrams displaying the overlap of downregulated proteins in the Model group that were reversed (upregulated) by EA treatment (top), and upregulated proteins in the Model group that were reversed (downregulated) by EA treatment (bottom). **(D)** Hierarchical clustering heatmap showing the relative expression levels (Z-score) of key overlapping DEPs among CON, HY, and EA groups (*n* = 3 samples per group). **(E)** Sankey pathway–protein linkage map depicting the connections between selected DEPs and their enriched KEGG pathways color-coded by log2 FC. **(F)** Bubble plot of top KEGG pathway enrichment analysis, with dot size representing gene count and color intensity indicating −log_(*p*-value). **(G)** Gene Ontology (GO) enrichment analysis of DEPs across Molecular Function (MF), Cellular Component (CC), and Biological Process (BP) categories, showing the top 20 enriched terms for each category based on gene count and adjusted *p*-value.

Cluster analysis (Figure 5D) revealed that mitochondrial-associated proteins such as Ndufa4, Atp5f1b, Cox4i1, Slc25a5, and Atp5f1a were significantly downregulated in the model group but upregulated following EA treatment. These proteins are primarily involved in the mitochondrial electron transport chain, ATP synthesis, and oxidative phosphorylation, suggesting that high-salt-induced hypertension is associated with impaired energy metabolism in the PVN, whereas EA may restore mitochondrial function. In contrast, inflammation- and stress-related proteins including S100b, Mif, Ppp3r1, Ptgds, Ywhaz, and Cfl1 were upregulated in the model group but significantly reduced after EA treatment. Among these, S100b, Mif, and Ptgds showed the most pronounced changes, indicating enhanced neuroinflammation and glial activation under hypertensive conditions, which were attenuated by EA. Overall, these findings suggest that EA reverses hypertension-induced proteomic abnormalities in the PVN, primarily through restoration of mitochondrial oxidative phosphorylation and suppression of neuroinflammatory pathways.

To further elucidate the biological functions and signaling pathways associated with differentially expressed proteins in the PVN, pathway–protein interaction networks were constructed (Figures 5E,F). Key proteins such as Atp5f1a, Atp5f1b, Cox4i1, and Ndufa4 served as central nodes converging on the oxidative phosphorylation pathway. Their upregulation in the EA group was closely associated with improvements in metabolic pathways, suggesting that EA enhances mitochondrial electron transport efficiency and alleviates metabolic suppression induced by hypertension. Notably, a substantial number of differentially expressed proteins were associated with neurodegenerative disease pathways, including Alzheimer's disease, Parkinson's disease, and Huntington's disease. Among them, Slc25a4 and Slc25a5 exhibited marked expression changes, suggesting systemic regulation of ATP/ADP exchange balance in PVN neurons under stress conditions. In addition, proteins such as S100b and Cfl1 were linked not only to neuronal injury responses but also to oxidative stress-related pathways. These findings indicate that the protective effects of EA on the PVN are not mediated by a single molecular target but rather through coordinated regulation of energy metabolism and oxidative stress defense networks. GO enrichment analysis (Figure 5G) further demonstrated that differentially expressed proteins were mainly enriched in transmembrane transport processes involving protons, ATP/ADP, and nucleotides, suggesting altered mitochondrial energy metabolism and membrane transport homeostasis in the PVN under hypertensive conditions.

At the molecular function level, the most significantly enriched terms included proton transmembrane transporter activity, ADP/ATP transmembrane transporter activity, and ATP synthase activity, along with the transport of purine nucleotides and carbohydrate derivatives. At the cellular component level, proteins were primarily localized to transmembrane transporter complexes and mitochondrial membrane structures. At the biological process level, enrichment was mainly associated with proton and nucleotide transport, ATP synthesis, intracellular pH regulation, and energy metabolism. Collectively, these results suggest that mitochondrial energy metabolism and transmembrane transport processes are significantly disrupted in the PVN during high-salt-induced hypertension. EA intervention may partially reverse these abnormalities by modulating these pathways and restoring metabolic homeostasis.

In summary, this study identifies mitochondrial energy metabolism imbalance in the PVN as a key pathological mechanism in hypertension and suggests that the therapeutic effects of EA may be closely associated with the regulation of proton and nucleotide transport and restoration of mitochondrial function.

### 3.6 Electroacupuncture restores ANT1 expression in the PVN of high-salt-induced hypertension mice

Given that proteomic analysis of the PVN revealed marked alterations in mitochondrial energy metabolism, we next focused on ANT1, encoded by SLC25A4, as a candidate protein potentially associated with the metabolic effects of EA. ANT1 is an integral protein of the mitochondrial inner membrane that plays an important role in the exchange of mitochondrial ATP for cytosolic ADP, thereby contributing to the maintenance of cellular energy homeostasis. In our proteomic dataset, ANT1 was among the mitochondrial-associated proteins showing altered expression in high-salt-induced hypertensive mice. In particular, ANT1 expression was reduced in the model group compared with the control group, whereas EA treatment increased ANT1 expression. This expression pattern was consistent with broader proteomic alterations involving oxidative phosphorylation, ATP metabolism, and mitochondrial nucleotide transport, suggesting that ANT1 may be associated with the mitochondrial metabolic remodeling observed in the PVN under high-salt conditions.

To independently validate the proteomic findings, we first examined ANT1 protein expression in the PVN by Western blotting. Consistent with the proteomic analysis, ANT1 protein levels were lower in the PVN of high-salt-induced hypertensive mice than in control mice. Following 3 weeks of EA at Taichong (LR3), ANT1 protein expression was significantly increased compared with that in untreated model mice, approaching the level observed in control mice. Quantification of the ANT1/β-actin ratio confirmed significant differences among the experimental groups (Figures 6A,C). These findings independently validated the proteomic results and indicated that the reduction in PVN ANT1 protein expression associated with high-salt-induced hypertension was accompanied by restoration following EA treatment.

**Figure 6:**
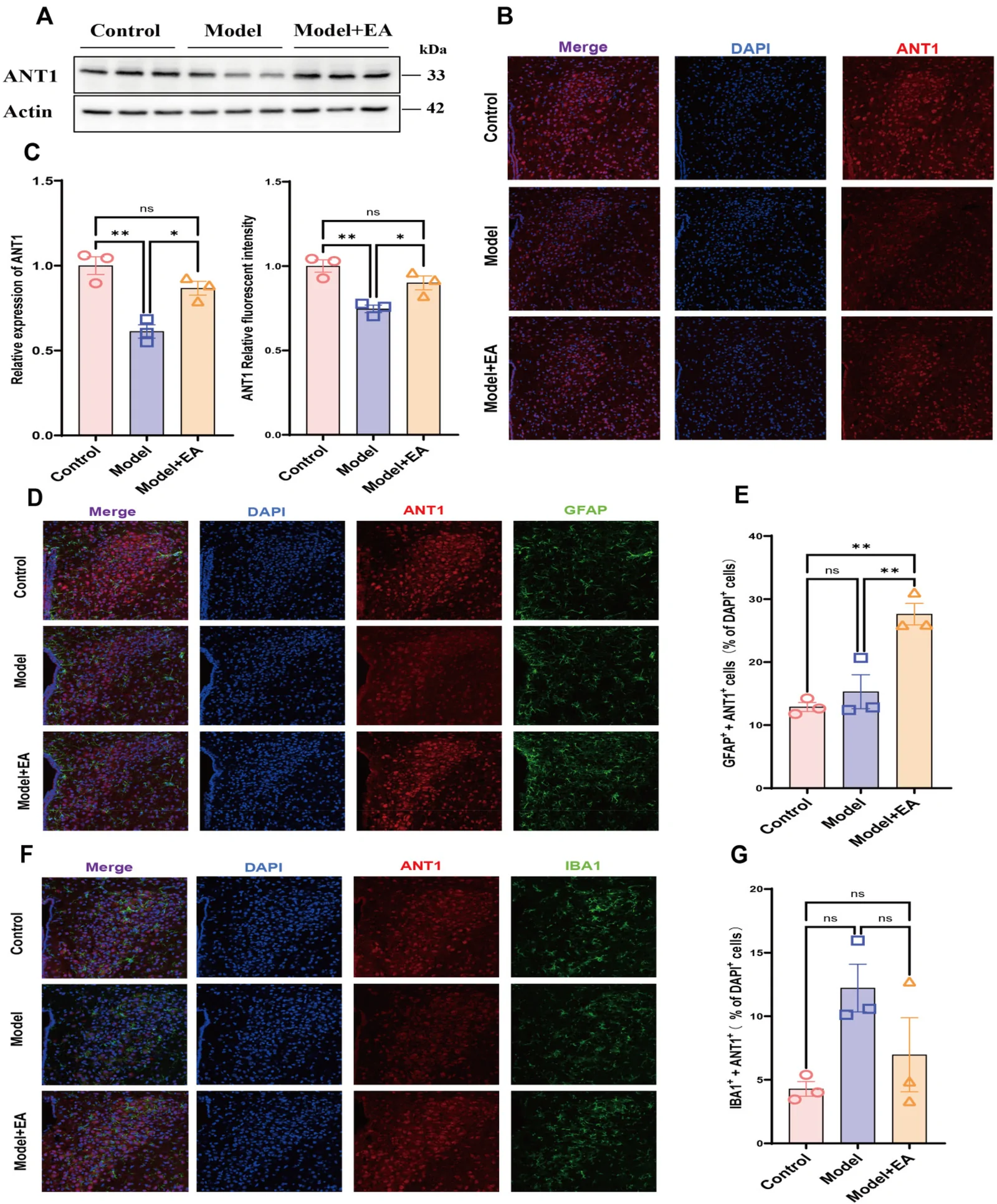
Electroacupuncture restores ANT1 expression in the PVN of high-salt-induced hypertension mice. **(A,C)** Western blot analysis of PVN tissues **(A)** and quantitative column graphs (C, left) showing the protein levels of ANT1 normalized to Actin across the Control, Model, and Model + EA groups, along with the corresponding quantitative evaluation of ANT1 relative fluorescence intensity in the PVN (C, right). **(B)** Representative immunofluorescence images showing ANT1 expression (red) co-stained with DAPI (blue) in the PVN across groups. **(D,E)** Representative immunofluorescence colabeling in the PVN **(D)** and quantitative percentage of GFAP⁺ ANT1⁺ cells among total DAPI⁺ cells **(E) (F,G)** Representative immunofluorescence colabeling in the PVN **(F)** and quantitative percentage of IBA1⁺ ANT1⁺ cells among total DAPI⁺ cells **(G)**. Data are presented as mean ± SEM (*n* = 3 per group). *p < 0.05,**p < 0.01, ***p < 0.001, ****p < 0.0001; ns, not significant.

We next performed immunofluorescence staining to further characterize the distribution of ANT1 within the PVN. ANT1 immunoreactivity was readily detectable in the PVN and exhibited a predominantly cytoplasmic/perinuclear staining pattern, consistent with its localization as a mitochondrial inner membrane protein. Compared with control mice, high-salt-induced hypertensive mice exhibited reduced ANT1 immunoreactivity in the PVN. In contrast, EA treatment increased ANT1 fluorescence intensity compared with that in the model group (Figures 6B,C). Quantitative analysis further showed that the proportion of ANT1-positive cells relative to DAPI-positive cells was reduced following high-salt exposure and partially restored by EA treatment. These histological findings were consistent with the changes detected by Western blotting and provided complementary evidence that ANT1 expression is altered in the PVN under conditions of high-salt-induced hypertension.

To further assess the cellular distribution of ANT1 within different cell populations in the PVN, we performed double immunofluorescence staining for ANT1 and the glial markers GFAP and IBA1. ANT1 immunoreactivity was detected in both GFAP-positive/ANT1-positive and IBA1-positive/ANT1-positive cells in the PVN, indicating that ANT1 immunoreactivity is present, at least in part, in astrocyte- and microglia-associated cell populations (Figures 6D,F). Quantitative analysis showed that the proportion of GFAP⁺/ANT1⁺/DAPI⁺ cells did not differ significantly between the model and control groups but was significantly increased following EA treatment. In contrast, no significant differences in the proportion of IBA1⁺/ANT1⁺/DAPI⁺ cells were observed among the experimental groups (Figures 6E,G).

It should be noted that NeuN staining was not sufficiently reliable for quantitative assessment of neuronal ANT1 expression because NeuN immunoreactivity is relatively weak in some PVN neurons, particularly magnocellular neurons. Therefore, we did not perform quantitative analysis of neuronal ANT1 expression based on NeuN colocalization. Nevertheless, considering the spatial distribution of ANT1 immunoreactivity together with the GFAP and IBA1 colocalization findings, ANT1 immunoreactivity appeared to be predominantly distributed within neuronal-enriched regions of the PVN, whereas only a subset of ANT1 signals overlapped with astrocytic or microglial markers. Thus, the present findings support the possibility that PVN neurons may represent the predominant cellular source of ANT1, while astrocytes and microglia may contribute to a lesser extent to the overall ANT1 expression in the PVN. However, because reliable quantitative assessment of neuronal ANT1 colocalization was not achieved in the present study, the precise relative contribution of different cell populations to ANT1 expression remains to be determined.

## 4 Discussion

This study demonstrates that electroacupuncture at Taichong (LR3) significantly reduces blood pressure and improves neurobehavioral impairments in salt-sensitive hypertensive mice. Moreover, EA modulates the excitability of PVN–RVLM and PVN–NTS projection neurons and reshapes the PVN proteomic landscape, with mitochondrial ANT1 emerging as a potential molecular target. These findings suggest that the antihypertensive effects of EA are closely associated with restoration of autonomic circuit homeostasis and regulation of mitochondrial-related pathways within the PVN.

High-salt-induced hypertension is characterized not only by sustained elevation of arterial pressure but also by profound alterations in central autonomic regulation and behavioral dysfunction (31, 32). Increasing evidence suggests that hypertension is frequently accompanied by anxiety, depression, and cognition-related abnormalities (33–36), largely attributable to neuroinflammation, oxidative stress, and neuronal dysfunction in central cardiovascular regulatory regions (7, 32, 37). In this study, long-term 2% NaCl intake successfully induced both hypertension and behavioral alterations, supporting the concept that high-salt-induced hypertension is a systemic disorder involving both cardiovascular and central nervous system dysfunction. Hypertension is highly prevalent worldwide, and salt sensitivity is observed in approximately 50% of individuals with hypertension and 25% of normotensive individuals (5). Despite its high prevalence, the mechanisms underlying salt sensitivity remain incompletely understood. Therefore, we selected a high-salt-induced hypertension model to investigate the neural mechanisms associated with salt-induced blood pressure elevation. Previous studies have demonstrated that male C57BL/6J mice exposed to a high-salt diet develop a significant increase in blood pressure (38, 39), consistent with our findings. This hypertensive response is accompanied by enhanced sympathetic activity and vascular reactivity, supporting the suitability of C57BL/6J mice for investigating neural and autonomic mechanisms associated with salt-induced blood pressure elevation (5, 38). These findings provided a rationale for selecting this model in the present study.

Nevertheless, several limitations of this model should be considered. First, the sodium load in our model is relatively high and was continuously administered through drinking water, which may not fully recapitulate the pattern and magnitude of sodium exposure encountered in humans. Second, the 4-week exposure period used in the present study primarily represents an early stage of salt-induced blood pressure elevation and may not reproduce the progressive renal, vascular, cardiac, and other end-organ remodeling associated with chronic hypertension. Third, high-salt exposure may directly influence renal sodium handling, vascular function, inflammatory signaling, and neural activity independently of changes in blood pressure (40–43). Therefore, the alterations in PVN neuronal excitability and mitochondrial-related proteins observed in the present study cannot be attributed exclusively to elevated blood pressure. Finally, because salt sensitivity is influenced by genetic background, sex, age, potassium intake, and other physiological factors (44), the present 2% NaCl paradigm should be interpreted as a model of high-salt-induced hypertension rather than a comprehensive representation of human salt-sensitive hypertension.

The PVN serves as a key integrative center for autonomic and neuroendocrine regulation and plays a central role in the pathophysiology of hypertension. Anatomically, the PVN projects directly to both the RVLM (9) and the NTS (45), thereby coordinating sympathetic outflow and baroreflex function. Enhanced excitability of PVN–RVLM presympathetic neurons has been well established as a major contributor to sympathetic overactivation and elevated blood pressure. In contrast, although anatomical studies have identified direct PVN–NTS projections as part of the central cardiovascular autonomic network (45), whether selective activation or inhibition of this pathway directly influences blood pressure has yet to be experimentally determined. In the present study, our electrophysiological recordings demonstrated that both PVN–RVLM and PVN–NTS projecting neurons exhibited significantly increased firing activity in hypertensive mice. Importantly, electroacupuncture effectively reversed the hyperexcitability of both PVN–RVLM and PVN–NTS projecting neurons, suggesting that modulation of PVN projection neuron excitability may contribute to the regulation of blood pressure by electroacupuncture.

Although previous studies have demonstrated that increased excitability of PVN–RVLM neurons represents an important upstream driver of sympathetic overactivation in hypertension (37), this finding does not directly demonstrate an increase in sympathetic nerve activity *in vivo*. Similarly, altered excitability of PVN–NTS projection neurons should not be interpreted as direct evidence of changes in parasympathetic output, particularly because the functional role of this pathway in cardiovascular regulation remains incompletely understood. Therefore, the effects of electroacupuncture observed in the present study should be interpreted as modulation of PVN projection neuron excitability rather than direct evidence of altered sympathetic or parasympathetic activity. Future studies combining *in vivo* measurements of sympathetic nerve activity, vagal activity, heart rate variability, or baroreflex sensitivity with selective manipulation of the PVN–RVLM and PVN–NTS pathways will be necessary to determine whether electroacupuncture-induced changes in PVN projection neuron excitability are causally linked to alterations in autonomic output and blood pressure regulation.

The mechanisms by which EA regulates central autonomic circuits remain incompletely understood. Recent studies highlight the importance of mitochondrial dysfunction and oxidative stress in neurogenic hypertension (46, 47). Mitochondria are essential for neuronal energy metabolism and excitability, and mitochondrial impairment leads to excessive ROS production, altered synaptic transmission, and sympathetic overactivation (48, 49). Our proteomic analysis revealed significant alterations in mitochondrial-related proteins, with ANT1 identified as a candidate protein. ANT1 (SLC25A4), a mitochondrial inner membrane protein, mediates ADP/ATP exchange and is essential for maintaining mitochondrial bioenergetics and cellular homeostasis.

Interestingly, ANT1 expression was significantly reduced in the PVN of hypertensive mice but was restored following EA treatment. Although the precise role of ANT1 in neurogenic hypertension remains to be fully elucidated, previous studies have associated reduced ANT1 expression with mitochondrial dysfunction, increased oxidative stress, and neuronal injury (20, 46, 50). We therefore speculate that ANT1 downregulation may contribute to neuronal dysfunction and autonomic imbalance in hypertension, whereas EA may exert beneficial effects at least in part by restoring ANT1-mediated mitochondrial homeostasis. However, the present study did not establish a causal relationship between ANT1 and the electrophysiological effects of electroacupuncture. Therefore, future studies using targeted manipulation of SLC25A4/ANT1, combined with electrophysiological and mitochondrial functional assessments, will be necessary to determine its causal role in the regulation of PVN neuronal excitability and the response to electroacupuncture.

Importantly, in this study, the proteomic findings were independently validated by Western blotting and immunofluorescence analyses. ANT1 protein expression and immunoreactivity were reduced in the PVN of high-salt-induced hypertensive mice and were partially restored following EA treatment at LR3. This parallel change across proteomic, biochemical, and histological measurements strengthens the evidence that ANT1 is associated with the metabolic alterations occurring in the PVN during high-salt-induced hypertension and their modulation by EA. Notably, the restoration of ANT1 expression occurred in parallel with the broader recovery of mitochondrial metabolism-related proteins identified by proteomic analysis. Although these findings do not establish that ANT1 directly mediates the metabolic effects of EA, they identify ANT1 as a potential molecular component associated with the restoration of PVN mitochondrial metabolic homeostasis following EA.

The cellular distribution of ANT1 also provides preliminary insight into its potential cellular context within the PVN. ANT1 immunoreactivity was detected in astrocyte- and microglia-associated populations, indicating that glial cells may contribute to the overall ANT1 signal detected in the PVN. However, only a subset of ANT1 immunoreactivity was associated with GFAP- or IBA1-positive cells, whereas ANT1 staining was predominantly observed in neuronal-enriched regions of the PVN. These observations raise the possibility that PVN neurons represent a major cellular source of ANT1, with astrocytes and microglia contributing to a lesser extent. Nevertheless, this interpretation should be considered cautiously because NeuN immunoreactivity was insufficiently reliable for quantitative assessment of ANT1 colocalization in some PVN neurons, particularly magnocellular neurons (51). Thus, the present data do not definitively establish the predominant cellular source of ANT1, and cell-type-specific approaches will be required to resolve its precise cellular distribution.

Taken together, our findings suggest that the reduction of ANT1 in the PVN may form part of the mitochondrial metabolic alterations associated with high-salt-induced hypertension, whereas its restoration following EA may reflect recovery of mitochondrial energy metabolism. However, the present study demonstrates an association rather than a causal relationship between ANT1 and the effects of EA. Whether ANT1 is required for EA-induced restoration of mitochondrial metabolism, PVN neuronal function, or cardiovascular regulation remains unknown. Future studies using cell-type-specific SLC25A4 loss- or gain-of-function approaches will be important to determine whether ANT1 is a necessary molecular mediator of the metabolic and cardiovascular effects of EA.

Electroacupuncture have been increasingly investigated as complementary therapeutic approaches for cardiovascular diseases, particularly hypertension and coronary heart disease. Clinical evidence suggests that acupuncture-based interventions may exert beneficial effects on cardiovascular disease. For example, a randomized controlled clinical trial reported that 8 weeks of electroacupuncture at selected acupoints significantly reduced systolic and diastolic blood pressure in patients with hypertension (52, 53). Notably, animal studies suggest that the reduction in blood pressure may be accompanied by decreases in plasma norepinephrine, renin, and aldosterone levels, suggesting that neurohumoral mechanisms related to cardiovascular regulation may contribute to the effects of electroacupuncture (54, 55). However, high-quality human clinical trials directly demonstrating this effect are currently lacking. In addition to hypertension, acupuncture has also been investigated as an adjunctive treatment for coronary heart disease and angina pectoris. Clinical trials and meta-analyses have reported potential improvements in angina-related outcomes when acupuncture is used in combination with conventional treatment (56). Similarly, randomized clinical studies in patients with heart failure have explored acupuncture or electroacupuncture as an adjunct to conventional treatment, with reported effects on cardiovascular and functional outcomes. In particular, trials have demonstrated improvements in left ventricular ejection fraction (LVEF), 6-minute walk distance, and quality of life, along with reductions in NT-proBNP levels and sympathetic activity (57, 58). While large-scale cohort data suggest a potential survival benefit (59), further high-quality RCTs focusing on hard endpoints like mortality and hospitalization are warranted to solidify these findings.

These clinical observations provide an important translational context for our findings. Although the present study does not directly establish the clinical efficacy of electroacupuncture, our results provide preclinical evidence that electroacupuncture may influence central neural mechanisms involved in cardiovascular regulation. In particular, we found that electroacupuncture modulated the intrinsic excitability of PVN projection neurons and was associated with changes in PVN proteomic signatures in high-salt-induced hypertension mice. These findings may help provide a mechanistic framework linking the cardiovascular effects observed in clinical acupuncture studies with alterations in central neural circuits involved in blood pressure regulation.

In conclusion, this study demonstrates that EA at LR3 alleviates high-salt-induced hypertension and associated behavioral alterations. These effects are accompanied by restoration of PVN–RVLM and PVN–NTS neuronal activity and remodeling of mitochondrial-related proteomic networks in the PVN. ANT1 may represent a novel molecular target involved in the central effects of EA, providing new insights into potential therapeutic strategies for neurogenic hypertension.

## Data Availability

The original contributions presented in this study are included in this article/supplementary material, further inquiries can be directed to the corresponding authors.

## References

[1] Momtazmanesh S Moghaddam SS Ghamari S-H Rad EM Rezaei N Shobeiri P. Global burden of chronic respiratory diseases and risk factors, 1990–2019: an update from the global burden of disease study 2019. eClinicalMed. (2023) 59:101936. 10.1016/j.eclinm.2023.101936 PMC7614570 37229504

[2] Chakraborty S Tiwari V Banerjee SK. Targeting the non-neuronal cholinergic machinery: a novel approach to mitigate cardiac aging. Ageing Res Rev. (2025) 109:102782. 10.1016/j.arr.2025.102782 40412764

[3] Farrar J Frieden T. WHO global report on hypertension 2025. Lancet. (2025) 406(10517):2318–9. 10.1016/S0140-6736(25)02208-1 41241506

[4] Chen X Zhong F Li J. The burden of cardiovascular disease attributable to dietary risk factors in China, 1990–2021. Sci Rep. (2025) 15(1):25641. 10.1038/s41598-025-11645-z 40664912 PMC12264015

[5] Feng Earley Y Pan S Verma H Zheng H Plata AA Zubcevic J. Central nervous system mechanisms of salt-sensitive hypertension. Physiol Rev. (2025) 105(4):1989–2032. 10.1152/physrev.00035.2024 40315132 PMC12187566

[6] Rasiah NP Loewen SP Bains JS. Windows into stress: a glimpse at emerging roles for CRHPVN neurons. Physiol Rev. (2023) 103(2):1667–91. 10.1152/physrev.00056.2021 36395349

[7] Zhang H Carter C Augenreich MA Kang X Liu Z Davis DJ. Unrevealing the role of hypothalamic corticotropin-releasing hormone neurons in blood pressure regulation in hypertension. Cardiovasc Res. (2025) 121(16):2565–77. 10.1093/cvr/cvaf211 41206456 PMC12713627

[8] Wang L Savani RH Lu Y Bernabucci M Luis-Islas J Park E. State-dependent central synaptic regulation by GLP-1 is essential for energy homeostasis. Nature Metabolism. (2025) 7(7):1443–58. 10.1038/s42255-025-01305-x 40467923 PMC12967307

[9] Ikeda S Shinohara K Ono Y Nakashima H Miyamoto R Hara A. Afferent renal nerves drive sympathoexcitation and hypertensive heart failure. Hypertension. (2026) 83(3):e25247. 10.1161/HYPERTENSIONAHA.125.25247 41498140

[10] Katayama PL Leirão IP Kanashiro A Luiz JPM Cunha FQ Navegantes LCC. The carotid body detects circulating tumor necrosis factor-alpha to activate a sympathetic anti-inflammatory reflex. Brain Behav Immun. (2022) 102:370–86. 10.1016/j.bbi.2022.03.014 35339628

[11] Ali MSS Parastooei G Raman S Mack J Kim YS Chung M-K. Genetic labeling of the nucleus of tractus solitarius neurons associated with electrical stimulation of the cervical or auricular vagus nerve in mice. Brain Stimul. (2024) 17(5):987–1000. 10.1016/j.brs.2024.08.007 39173736 PMC11555405

[12] Lu M-X Huang J-Y Xu X-Z Sun J-Y Zou D-N Zhang J-Y. Parasubthalamic glutamatergic neurons coordinate cardiovascular homeostasis and locomotion in mice. Adv Sci. (2025) 12(40):e17353. 10.1002/advs.202417353 PMC12561448 40719193

[13] Liu J- Han B- Liu Y Nie B- Bian T Liu C. Electroacupuncture treatment can improve cognitive impairment in spontaneously hypertensive rats: a preliminary DTI study. Front Neurosci. (2025) 19:1637037. 10.3389/fnins.2025.1637037 40948815 PMC12423048

[14] Wang Y Chang X Zhang H Hou Y Zheng X Zhang Y. Hypothalamic gene expression in a rat model of chronic unpredictable mild stress treated with electroacupuncture. Neurochem Res. (2024) 49(5):1406–16. 10.1007/s11064-024-04124-w 38522048

[15] Zhang Q Tan Y Wen X Li F. Involvement of neuropeptide Y within paraventricular nucleus in electroacupuncture inhibiting sympathetic activities in hypertensive rats. Int J Hypertens. (2022) 2022:9990854. 10.1155/2022/9990854 35087687 PMC8789434

[16] Jiao S Yueming W Jian L. Effect of electroacupuncture on hypertensive and sympathetic excitability mechanism mediated by the paraventricular nucleus of the hypothalamus in spontaneous hypertensive rats. J Tradit Chin Med. (2025) 45(3):586–96. 10.19852/j.cnki.jtcm.2025.03.013 40524297 PMC12134317

[17] Tjen-A-Looi SC Fu L-W Guo Z-L Gong YD Nguyen ATN Nguyen A-TP. Neurogenic hypotension and bradycardia modulated by electroacupuncture in hypothalamic paraventricular nucleus. Front Neurosci. (2022) 16:934752. 10.3389/fnins.2022.934752 35958987 PMC9361000

[18] Zhang Z Zhao X Zhang X Wan S An P Zhu Y. Mitochondrial cardiovascular diseases: molecular mechanisms, multi-omics exploration and therapeutic strategies. J Adv Res. (2026) 82:347–65. 10.1016/j.jare.2025.07.021 40680831 PMC13001046

[19] Camargo LL Rios FJ Montezano AC Touyz RM. Reactive oxygen species in hypertension. Nat Rev Cardiol. (2025) 22(1):20–37. 10.1038/s41569-024-01062-6 39048744

[20] Zhao Y Shi F Hao H Wang Z Li J Liu B. Hypertension increases susceptibility to lead-induced microglial polarization via ANT1-mediated mitochondrial DNA/cGAS/STING signaling. Research. (2025) 8:1026. 10.34133/research.1026 41404489 PMC12703018

[21] Angelin A Keller K Lu P Guarnieri JW Widjaja GA Sule R. Partial restoration of mitochondrial dysfunction by AAV-Ant1 protects from dilated cardiomyopathy in Ant1-/- plus mtDNA mutant mice. Nat Commun. (2026) 17(1):447. 10.1038/s41467-025-67134-4 PMC12800341 41398158

[22] Carnovale C Perrotta C Baldelli S Cattaneo D Montrasio C Barbieri SS. Antihypertensive drugs and brain function: mechanisms underlying therapeutically beneficial and harmful neuropsychiatric effects. Cardiovasc Res. (2023) 119(3):647–67. 10.1093/cvr/cvac110 35895876 PMC10153433

[23] Mulligan MD Murphy R Reddin C Judge C Ferguson J Alvarez-Iglesias A. Population attributable fraction of hypertension for dementia: global, regional, and national estimates for 186 countries. eClinicalMed. (2023) 60:102012. 10.1016/j.eclinm.2023.102012 PMC10227413 37261323

[24] Chen W Luo Z Huang M Zheng X Xiong N Gan C. Targeting the 5-HT1A receptor in the ventral hippocampus alleviates anxiety-like behavior in mice with angiotensin II-induced hypertension. Cell Biosci. (2026) 16(1):72. 10.1186/s13578-026-01583-2 42093043 PMC13339978

[25] Lin GH Chang WC Chen KJ Tsai CC Hu SY Chen LL. Effectiveness of acupressure on the taichong acupoint in lowering blood pressure in patients with hypertension: a randomized clinical trial. Evid Based Complement Alternat Med. (2016) 2016:1549658. 10.1155/2016/1549658 27803727 PMC5075632

[26] Wei B Cheng G Bi Q Lu C Sun Q Li L. Microglia in the hypothalamic paraventricular nucleus sense hemodynamic disturbance and promote sympathetic excitation in hypertension. Immunity. (2024) 57(9):2030–2042.e8. 10.1016/j.immuni.2024.07.011 39116878

[27] Gottlieb LA Mahfoud F Stavrakis S Jespersen T Linz D. Autonomic nervous system: a therapeutic target for cardiac end-organ damage in hypertension. Hypertension. (2024) 81(10):2027–37. 10.1161/HYPERTENSIONAHA.123.19460 39136127 PMC11404762

[28] Mowry FE Peaden SC Stern JE Biancardi VC. TLR4 and AT1R mediate blood-brain barrier disruption, neuroinflammation, and autonomic dysfunction in spontaneously hypertensive rats. Pharmacol Res. (2021) 174:105877. 10.1016/j.phrs.2021.105877 34610452 PMC8648989

[29] Cao W Yang Z Liu X Ren S Su H Yang B. A kidney-brain neural circuit drives progressive kidney damage and heart failure. Signal Transduct Target Ther. (2023) 8(1):184. 10.1038/s41392-023-01402-x 37169751 PMC10175540

[30] Wang X Yue Z Shi L He W Shao L Liu Y. Activation of centromedial amygdala GABAergic neurons produces hypotension in mice. Neurosci Bull. (2025) 41(5):759–74. 10.1007/s12264-024-01317-9 39581900 PMC12014883

[31] Su Q Yu X-J Wang X-M Peng B Bai J Li H-B. Na+/K+-ATPase alpha 2 isoform elicits Rac1-dependent oxidative stress and TLR4-induced inflammation in the hypothalamic paraventricular nucleus in high salt-induced hypertension. Antioxidants. (2022) 11(2):288. 10.3390/antiox11020288 35204171 PMC8868219

[32] Stocker SD. Altered neuronal discharge in the organum vasculosum of the lamina terminalis contributes to dahl salt-sensitive hypertension. Hypertension. (2023) 80(4):872–81. 10.1161/HYPERTENSIONAHA.122.20798 36752103 PMC10023399

[33] Yang L Ao Y Li Y Dai B Li J Duan W. Morinda officinalis oligosaccharides mitigate depression-like behaviors in hypertension rats by regulating Mfn2-mediated mitophagy. J Neuroinflammation. (2023) 20(1):31. 10.1186/s12974-023-02715-y 36765376 PMC9912533

[34] Nist KM Bard H McBride B Capriglione AL Moreira JD Farb DH. Losartan attenuates sex-dependent hypertension, neuroinflammation, and cognitive impairment in the aging male sprague-dawley rat. GeroScience. (2025) 47(3):3007–26. 10.1007/s11357-024-01409-4 39627570 PMC12181475

[35] Kubota H Kunisawa K Wulaer B Hasegawa M Kurahashi H Sakata T. High salt induces cognitive impairment via the interaction of the angiotensin II-AT1 and prostaglandin E2-EP1 systems. Br J Pharmacol. (2023) 180(18):2393–411. 10.1111/bph.16093 37076133

[36] Zhang J Xie C Xu P Tong Q Xiao L Zhong J. Projections from subfornical organ to bed nucleus of the stria terminalis modulate inflammation-induced anxiety-like behaviors in mice. Sci Adv. (2024) 10(48):eadp9413. 10.1126/sciadv.adp9413 39602546 PMC11601211

[37] Bi Q Wang C Cheng G Chen N Wei B Liu X. Microglia-derived PDGFB promotes neuronal potassium currents to suppress basal sympathetic tonicity and limit hypertension. Immunity. (2022) 55(8):1466–1482.e9. 10.1016/j.immuni.2022.06.018 35863346

[38] Ralph AF Grenier C Costello HM Stewart K Ivy JR Dhaun N. Activation of the sympathetic nervous system promotes blood pressure salt-sensitivity in C57BL6/J mice. Hypertension. (2021) 77(1):158–68. 10.1161/HYPERTENSIONAHA.120.16186 33190558 PMC7720873

[39] Sabouri M Zheng X Irwin BJ Machin DR. Effects of excess sodium consumption on arterial function in C57BL/6 mice. Am J Physiol Heart Circ Physiol. (2024) 327(4):H896–907. 10.1152/ajpheart.00242.2024 39150393 PMC11482244

[40] Torimitsu T Kinouchi K Changizi Ashtiani K Abdelkarim S Sato T Kozuma T. Circadian reprogramming by timed sodium intake reveals transcriptional pathways of daily salt handling in the colon. Sci Adv. (2026) 12(11):eady9244. 10.1126/sciadv.ady9244 41824567 PMC12985676

[41] Nascimento AF Luvizotto RAM Costa RM Pimenta GF Bruder A Bruder-Nascimento T. High-salt diet-induced endothelial dysfunction is mediated by cellular senescence. J Am Heart Assoc. (2025) 40:1282. 10.1152/physiol.2025.40.S1.1282 PMC13279259 41988978

[42] Ahmad T Saleem M Mutchler AP Ertuglu LA Sheng Q Albritton CF. Myeloid activator protein-1 complex contributes to salt-sensitive hypertension. Circ Res. (2026) 139(7):e327976. 10.1161/CIRCRESAHA.125.327976 42605627 PMC13543672

[43] Nomura K Hiyama TY Sakuta H Matsuda T Lin C-H Kobayashi K. [Na+] increases in body fluids sensed by central nax induce sympathetically mediated blood pressure elevations via H+-dependent activation of ASIC1a. Neuron. (2019) 101(1):60–75.e6. 10.1016/j.neuron.2018.11.017 30503172

[44] Todua I. Management of salt-sensitive hypertension in clinical settings: how should we approach it? Am J Med. (2026) 139(10):1286–93. 10.1016/j.amjmed.2026.05.003 42103091

[45] Singh U Jiang J Saito K Toth BA Dickey JE Rodeghiero SR. Neuroanatomical organization and functional roles of PVN MC4R pathways in physiological and behavioral regulations. Mol Metab. (2022) 55:101401. 10.1016/j.molmet.2021.101401 34823066 PMC8689242

[46] Wang L Liu T Wang X Tong L Chen G Zhou S. Microglia-derived TNF-α contributes to RVLM neuronal mitochondrial dysfunction via blocking the AMPK-Sirt3 pathway in stress-induced hypertension. J Neuroinflammation. (2023) 20(1):137. 10.1186/s12974-023-02818-6 37264405 PMC10236846

[47] Kalsoom U-E Jin J Meng W Su Y Wu C Wu Q. The role of NADPH oxidases in central nervous system regulation of hypertension: mechanisms and therapeutic insights. Biomed Pharmacother. (2026) 196:119096. 10.1016/j.biopha.2026.119096 41653903

[48] Du M Wu C Yu R Cheng Y Tang Z Wu B. A novel circular RNA, circIgfbp2, links neural plasticity and anxiety through targeting mitochondrial dysfunction and oxidative stress-induced synapse dysfunction after traumatic brain injury. Mol Psychiatry. (2022) 27(11):4575–89. 10.1038/s41380-022-01711-7 35918398 PMC9734054

[49] Liu Z Shan S Kang K Wang S Yong H Sun Y. Mitochondrial transfer of *α*-synuclein mediates carbon disulfide-induced mitochondrial dysfunction and neurotoxicity. Ecotoxicol Environ Saf. (2024) 281:116613. 10.1016/j.ecoenv.2024.116613 38908057

[50] Ferrari F Villa RF. Brain bioenergetics in chronic hypertension: risk factor for acute ischemic stroke. Biochem Pharmacol. (2022) 205:115260. 10.1016/j.bcp.2022.115260 36179931

[51] Yu P McKinney EC Kandasamy MM Albert AL Meagher RB. Characterization of brain cell nuclei with decondensed chromatin. Dev Neurobiol. (2015) 75(7):738–56. 10.1002/dneu.22245 25369517

[52] Wang J- Yang M- Wu Q- Chen J Deng S- Chen L. Improvement of intestinal flora: accompany with the antihypertensive effect of electroacupuncture on stage 1 hypertension. Chin Med. (2021) 16(1):7. 10.1186/s13020-020-00417-8 33413552 PMC7792359

[53] Zhong X Yu C Guo H. Acupuncture-based interventions for blood pressure control and cardiovascular risk-related outcomes in adults with hypertension: a systematic review and meta-analysis of randomized controlled trials. Front Med (Lausanne). (2026) 13:1888399. 10.3389/fmed.2026.1888399 42656444 PMC13506824

[54] Wang X-R Yang J-W Ji C-S Zeng X-H Shi G-X Fisher M. Inhibition of NADPH oxidase-dependent oxidative stress in the rostral ventrolateral medulla mediates the antihypertensive effects of acupuncture in spontaneously hypertensive rats. Hypertension. (2018) 71(2):356–65. 10.1161/HYPERTENSIONAHA.117.09759 29229746

[55] Ye Z Zhu L Li X- Gao H- Wang J Wu S- PC6 electroacupuncture reduces stress-induced autonomic and neuroendocrine responses in rats. Heliyon. (2023) 9(4):e15291. 10.1016/j.heliyon.2023.e15291 37095918 PMC10121450

[56] Zhao L Li D Zheng H Chang X Cui J Wang R. Acupuncture as adjunctive therapy for chronic stable angina: a randomized clinical trial. JAMA Intern Med. (2019) 179(10):1388–97. 10.1001/jamainternmed.2019.2407 31355870 PMC6664382

[57] Leem J Lee SMK Park JH Lee S Chung H Lee JM. Efficacy and safety of electroacupuncture in acute decompensated heart failure: a study protocol for a randomized, patient- and assessor-blinded, sham controlled trial. BMC Complement Altern Med. (2017) 17(1):361. 10.1186/s12906-017-1864-3 28697773 PMC5504710

[58] Ilic D Jovic Z Mladenovic Z Pejovic V Lung B Kozic A. Acupuncture and kinesitherapy improve physical activity more than kinesitherapy alone in patients with acute decompensated chronic heart failure with reduced ejection fraction who are already on optimal drug therapy: a randomized, sham-controlled, double-blind clinical study. Biomedicines. (2025) 13(1):176. 10.3390/biomedicines13010176 39857763 PMC11759862

[59] Jun H Park D Sul J-U Cheong MJ Kim H Youn I. Impact of acupuncture on mortality in patients with disabilities and newly diagnosed heart failure: a nationwide cohort study. Front Med (Lausanne). (2025) 12:1519588. 10.3389/fmed.2025.1519588 39944496 PMC11813762

